# Comparison of stable isotope mixing models for examining plant root water uptake

**DOI:** 10.1371/journal.pone.0318771

**Published:** 2025-02-12

**Authors:** Xue Qiu, Mingjun Zhang, Shengjie Wang, Hongfei Meng, Cunwei Che

**Affiliations:** 1 College of Urban Environment, Lanzhou City University, Lanzhou, Gansu Province, China; 2 College of Geography and Environmental Science, Northwest Normal University, Lanzhou, Gansu Province, China; 3 Key Laboratory of Resource Environment and Sustainable Development of Oasis of Gansu Province, Northwest Normal University, Lanzhou, Gansu Province, China; 4 Laboratoire de Météorologie Dynamique, IPSL, CNRS, Sorbonne Université, Paris, France; 5 Northwest Institute of Eco-Environment and Resources, Chinese Academy of Sciences, Lanzhou, Gansu Province, China; Chinese Academy of Forestry, CHINA

## Abstract

As natural isotope tracers, *δ*^18^O and *δ*^2^H have been widely applied to examine the water uptake of plant root, but the various stable isotope mixing models may lead to different explanations. To understand the influence of models, here we selected a typical plant *Caragana korshinskii* Kom. in northeastern Tibetan Plateau, and analyzed the stable water isotope compositions in plant xylem and potential sources including precipitation and soil water. Three stable isotope mixing models, i.e., IsoSource, MixSIR and MixSIAR were used, and then the differences in the proportional contributions of various water sources for plant root were examined. The results showed that the IsoSource and MixSIR were generally similar, while the MixSIAR were significantly different. Although the proportional contributions of each water source were different due to different algorithms, the water source which contributed maximum proportion was the same for all models. This study provides a scientific reference for the selection of models for the study of plant water use strategies in similar study areas, i.e., the most dominant water source can be identified regardless of the choice of model.

## Introduction

Stable isotopes are an useful tool for ecologists and hydrologists, for determining water, carbon, nutrient and trace element fluxes and cycling in a variety of systems [1]. Using stable isotope tracer, the proportional contribution of several sources (such as water source) to a mixture (such as plant) can be determined in mixing models. Besides the diet analysis as an important application of stable isotope mixing models [2], there are many other cases of these mixing model, such as diagnosing the sources of water took up by plants [3–5], atmospheric or water pollution [6], groundwater recharge [7], precipitation nitrogen sources [8] and nitrate sources [9].

In recent years, a number of isotope-based mixing models have been developed to estimate proportional contribution of multiple sources to a mixture. A traditional linear mixing model, IsoSource, is designated based on mass balance, by which the proportional contributions of *n* + 1 different sources can be uniquely determined by the use of *n* different tracers, and find multiple combinations of source proportions which are feasible solutions [1,10].

Uncertainty and comparability within the mixing models are often non-negligible, and has been continuously discussed since the mixing model was applied. Bayesian-mixing model was firstly proposed to estimates probability distributions of source contributions to a mixture, and then developed MixSIR, SIAR and MixSIAR softwares. Bayesian mixing models improve upon linear mixing model by considering uncertainty associated with multiple sources, fractionation [11,12], categorical and continuous covariates [13], and these models also allow for optional incorporation of informative prior information in analyses [11].

Water in plants usually does not undergo isotope fractionation before being transported from roots to leaves [14], therefore the stable isotope information in plant xylem water can reflect the isotope information of its source water. As a result, the mixing model is introduced to convert isotope information into proportional contribution of plant water source [15–20]. Accordingly, a comprehensive comparison and analysis for different mixing models were conducted based on stable isotope data of plant xylem water and potential source water.

At present, the comparison studies on the application of various stable isotope mixing models to plant root water uptake are relatively limited, and it is not clear how different the various models are and which model is more suitable for individual research needs. There have been some comparative studies on different stable isotope mixing models [21–26]. For example, the uncertainties in plant water source partitioning of apple trees in the southern Loess Plateau were researched [21], four tracers of different characteristics, two xylem water deuterium bias correction methods, and four mixing models (IsoSource, SIAR, MixSIR, and MixSIAR) were combined to quantify the total uncertainty and the uncertainty of each component. The results showed that tracer combination of ^2^H and ^18^O with MixSIAR was the best framework for water source partitioning. Similarly, the differences among IsoSource and three Bayesian models (SIAR, MixSIR, MixSIAR) were examined, as well as the influence of single or dual isotope tracers to identify sources of water absorbed by *Vitex negundo*, *Sophora viciifolia* (shrubs) and *Artemisia gmelinii* (subshrub) during the growing season in the semiarid Loess Plateau [22]. The result showed that the SIAR and MixSIAR models exhibited relatively better water source apportionment performances than that of the MixSIR model. This is also supported by other similar studies. For example, the performance of MixSIAR model outperformed IsoSource model in estimating plants water sources in Karst areas (the main dominant plants in the secondary forest of southwest karst regions, *Kalopanax septemlobus* (Thunb*.*) Koidz., *Toona sinensis* and *Platycarya strobilacea* Sieb*. et* Zucc. were selected [23]. MixSIAR performed relatively better than other models in predicting root water uptake of winter wheat under three different soil moisture conditions in the North China Plain [24]. However, the findings are not consistent in various publications, indicating the integrated comparison under various climate and vegetation backgrounds are still needed.

In this study, we collected the samples of a typical plant and the potential water sources in northeastern margin of the Qinghai-Tibet Plateau and then compared the three mixing models using these samples. *Caragana korshinskii* Kom. (*C. korshinskii*) is a typical dominant plant in the sampling site and is a representative drought-tolerant plant, which can provide a scientific reference for the study of water use strategies of drought-tolerant plants in other similar study areas.

The purpose of this study is to: (1) provide a comprehensive summary of different stable isotope mixing model; (2) clarify the possible influence of model selection on the results; and (3) provide a reference for the model selection of root water uptake in similar cold and mountainous areas.

## Materials and methods

### Sample collection

**Fig 1:**
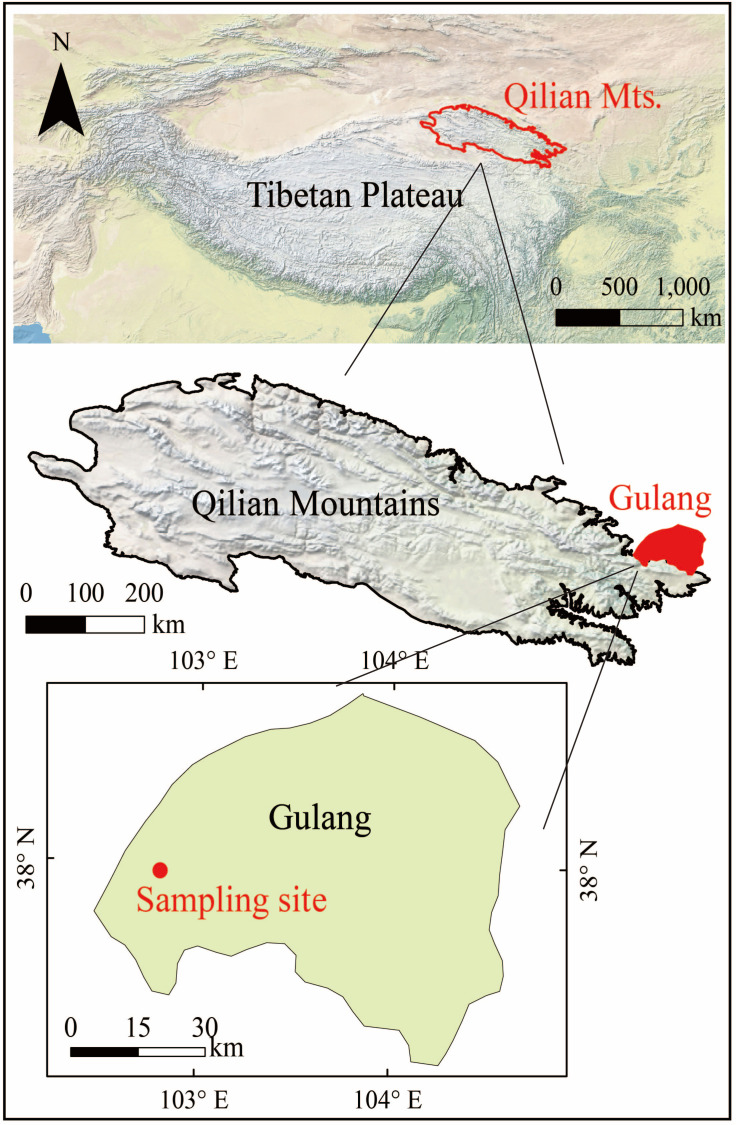
The location of the sampling point (map image from Natural Earth,http://www.naturalearthdata.com/).

*C. korshinskii* is a leguminous shrub belonging to the legume, 1 ~ 4 m high, its old branches are golden yellow and glossy, shoots are white pilose. The pinnately compound leaves have 6–8 pairs of leaflets, and the pods are flat and lanceolate. It is widely distributed in the northwest China and Mongolia. The plant stem of *C. korshinskii* (average plant height 1.4 m) and potential water source samples were collected in the Gulang County, Wuwei City, Gansu Province in China on May 16, 2017 (Table 1 and Fig 1). Fig 1 presented the location of the sampling point (102°54′E, 37°29′N, 2072.4 m a.s.l.), which located in the east of the Qilian Mountains, the largest marginal mountain in the northeastern Qinghai-Tibet Plateau. The Qilian Mountains located in the interior area and are the transition area between the northwest desert region and the Qinghai-Tibet alpine region. Therefore, it has both continental climate and plateau climate. The average annual air temperature of Gulang is 7.3°C, and the average annual precipitation is 463.8 mm. The combined effects of different topography, climate, hydrogeology, soil-forming materials and human activities have resulted in the formation of nine types of soils, including subalpine meadow soils, mountain gray-brown soils, mountain black-calcium soils, gray-calcium soils, laterite soils, wind-sand soils, oasis irrigation and silt soils, and tidal soils. The vegetation in the territory is characterized by diversity and has a horizontal and vertical distribution pattern, and is roughly divided into five vegetation zones: the mountain vertical zone, the mountain scrub-meadow vegetation zone, the mountain grassland vegetation zone, the mountain desert vegetation zone, and the desertified grassland vegetation zone in the plains (http://www.gulang.gov.cn/).

**Table 1:** Isotope data of water samples used for model comparison.

Sample type		Mean ± SD (%)		*N*
		*δ*^2^H (%)	*δ*^18^O (%)	
Plant stem	/	−56.12 ± 0.99	−7.20 ± 0.08	2
Water source	Soil, 0−20 cm	−50.48 ± 11.81	−4.84 ± 2.64	6
	Soil, 20−50 cm	−50.46 ± 2.12	−5.48 ± 0.47	8
	Soil, 50−70 cm	−48.60 ± 2.50	−5.65 ± 0.63	6
	Soil, 70−100 cm	−49.73 ± 2.16	−5.92 ± 0.41	8
	Precipitation two days before sampling *P* = 10.3 mm	−76.55	−11.67	1
	Precipitation one day before sampling *P* = 3.3 mm	−70.36	−10.79	1

About the basis for water source identification and division, since the sampling point is far from river, and the groundwater table depth exceeds the threshold value for plant water use, i.e., there is no well water or outcrop spring near the sampling point. Besides precipitation occurred one or two days before the sampling, so the potential water source used by plants were assumed to be precipitation and soil water. Regarding the division of soil layers, for the purpose of calculating, the 0–100 cm soil layers were divided into four soil layers based on soil water isotope characteristics. In order to eliminate the isotope enrichment caused by stomatal transpiration, plant stems were collected between 8:00 and 10:00 am Beijing Time when the evaporation is weak. The stems more than 2 years old and the branch segments with a diameter of 0.3 ~ 0.5 cm and a length of 3 ~ 5 cm were selected, then the outer bark and phloem of the branch segments were peeled off immediately and the reserved xylem segments were putted into sampling bottles and sealed.

Regarding the soil sampling, according to the roots distribution characteristics of *C. korshinskii*, roots were distributed vertically in the uppermost portion of the soil profile [27], especially the coarse roots, which were concentrated in the upper 0.4 m in the Tengger Desert [28]. In addition, *C. korshinskii* relied on water from the deep soil layer (80–100 cm) (mean 52%) during the dry period (May and June) on the Loess Plateau [27], and the soil-water content (SWC) varied little at depths > 100 cm in the Loess Plateau [29], soil water at a depth of 100 cm can thus provide isotopic information for deeper soil water. Thus soil profile (approximately 1 m ×  1 m ×  1 m) was dug at the sampling site, and soil samples at different depths were collected from bottom to top with intervals of 10 cm. Since the top soil was exposed to the air for a long time, the collection of the shallow soil started from 2 cm below the surface, then the samples were directly filled into sampling bottles and sealed. The final isotope value for each sample was an arithmetic average of several repeat samples.

### Laboratory analysis

Water was drawn from plant xylem and soil using the Automatic Water Extraction System LI-2100 (LICA United Technology Limited, China). Then the isotope compositions (^18^O and ^2^H) in liquid water including precipitation, soil water and xylem water were analyzed by the Liquid Water Isotope Analyzer DLT-100 (Los Gatos Re-search, USA) in the Stable Isotope Laboratory, College of Geography and Environmental Science, Northwest Normal University. More information of the analysis was detailed in another paper [30].

The isotope ratios of ^18^O/^16^O and ^2^H/^1^H in the water samples are expressed as *δ*^18^O and *δ*^2^H which are relative to the deviation of the ratios to the Vienna Standard Mean Ocean Water (V-SMOW).


$$\delta^{18}O=\left(\frac{R_{sample}}{R_{standard}}-1\right)\times 1000‰$$ (1)

$$\delta^{2}H=\left(\frac{R_{sample}}{R_{standard}}-1\right)\times 1000‰$$ (2)

where *R*_sample_ is the ratio of ^18^O/^16^O (^2^H/^1^H) in the water sample, and *R*_standard_ is the ratio of ^18^O/^16^O (^2^H/^1^H) in V-SMOW. The measurement accuracy for *δ*^18^O and *δ*^2^H is 0.2‰ and 0.6‰, respectively.

### Stable isotope mixing models

#### IsoSource.

IsoSource is a visual basic program to determine the relative proportion of each source to the mixture based on linear equations. The user inputs the isotopic signatures of the sources and the mixture, along with the suitable source increment and the mass balance tolerance. Output files include all the feasible source combinations, with histograms and descriptive statistics on the distributions for each source. The IsoSource program is available for public use at http://www.epa.gov/wed/ pages/models.htm [1].

A basic mass-balance mixing model assumes that, for a given isotope, the isotopic composition of the mixture (*δ*_M_) is defined as follows:


$$\begin{array}{l} \delta_{M}=f_{A}\delta_{A}+f_{B}\delta_{B}+f_{C}\delta_{C}\\ 1=f_{A}+f_{B}+f_{C} \end{array}$$ (3)

where *δ*_M_ is the isotope composition of mixture; *δ*_A,_ *δ*_B_ and *δ*_C_ are the isotope composition of sources, respectively; *f*_A_, *f*_B_, and *f*_C_ are the fractions of source A, B and C, respectively.

Given the ongoing proliferation of stable isotope studies, there is a clear need for robust analytical techniques that allow for the estimation of uncertainty surrounding source contributions.

#### MixSIR.

MixSIR is a graphical interface (GUI) program built on the Matlab platform that carries out Bayesian analysis of stable isotope mixing model using sampling-importance-resampling (SIR) [11]. The MixSIR GUI allows user to input isotope data and specify graphical and textual outputs of probability distributions for the source contributions, and it incorporate prior information and uncertainty into stable isotope mixing model. MixSIR is available over the GreenBoxes code sharing network (http://conserver.iugo-café.org), along with example data and a user guide with more information regarding the model form and function.

#### MixSIAR.

MixSIAR GUI is a Graphical User Interface that helps user build and run Bayesian mixing models to analyse biotracer data, and it following the MixSIAR model framework. Both the GUI version and script version are written in the open source languages R, then MixSIAR writes a custom JAGS (i.e., Just Another Gibbs Sampler) model file, runs the model in JAGS, and finally produces diagnostics, posterior plots, and summary statistics. What should be noted is that MixSIAR is based on MCMC (Markov chain Monte Carlo) chains, so before users accept the output, one should be able to confirm that the chain is converged by one of the two default methods for diagnosing the convergence of the model, namely Gelman-Rubin and Geweke diagnosis.

More information about MixSIAR GUI was introduced in paper [27], and most of the mathematical formulation underlying model and primary citation for Bayesian mixing models, refer to the paper [13].

Table 2 compared the basic information of each mixing model.

**Table 2:** Basic information of stable isotope mixing models (IsoSource, MixSIR and MixSIAR).

Model	Principle	Input data	Output data	Advantages	Disadvantages
IsoSource	Lineal mixing model	isotopic signatures of mixture and potential sources, increment, tolerance	“.tot” file, “.out” file; percent frequency of source proportion	Simple operation; Output all possible solution combinations	Up to 5 isotopes and 10 sources are allowed; Uncertainty is not taken into account
MixSIR	Bayesian mixing model	“mean_frac.txt”, “mean_source.txt”, “mix_data.txt”, “SD_frac.txt”, “SD_source.txt”	“contrib_out.txt”; posterior probability of source contribution	Uncertainty is taken into account; Output all feasible solution combinations; Allow for more sources and isotopes	Residual error is not considered
MixSIAR	Bayesian mixing model	mixture data, source data, discrimination data	“summary_statistics.txt”; scaled posterior density of source proportion	Consider both uncertainty and residual error; Allow for more sources and isotopes	Not all feasible solution combinations are listed; It takes a long time to convergence diagnose model

### ANOVA

Analysis of Variance (ANOVA), is used for significance tests of differences between the means of two or more samples. In this research, the ANOVA analysis was performed using SPSS software (IBM SPSS Statistics, version 25.0) to compare the differences in calculation results of three stable isotope mixing models.

### Mapping software

The mapping software used in this research include ArcGIS (version 10.7) and Sigma Plot (version 12.5).

## Results

**Fig 2:**
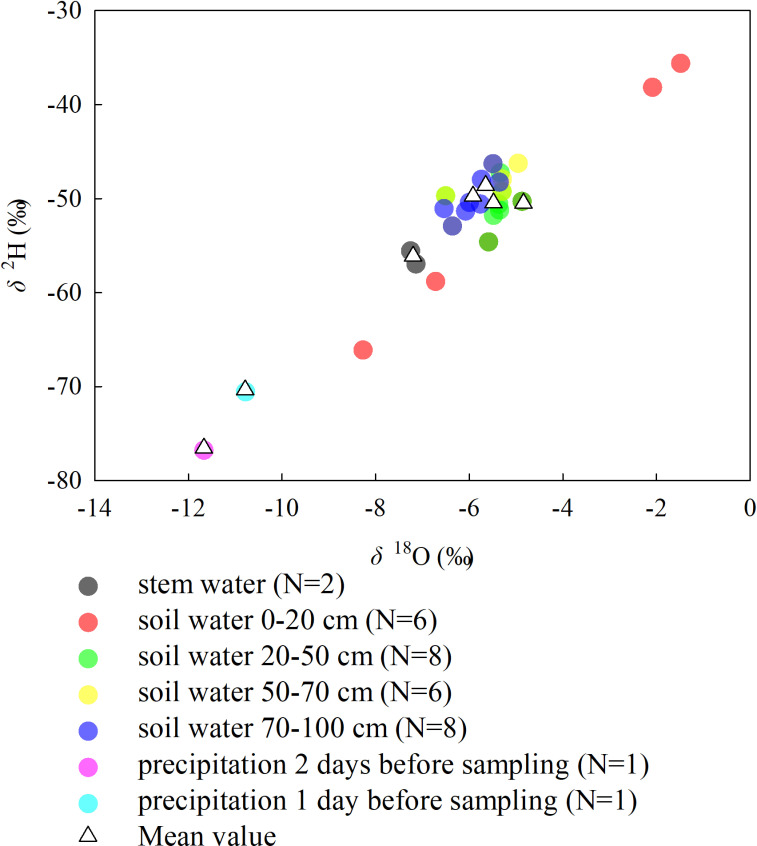
The dual isotope plot of the water samples.

**Fig 3:**
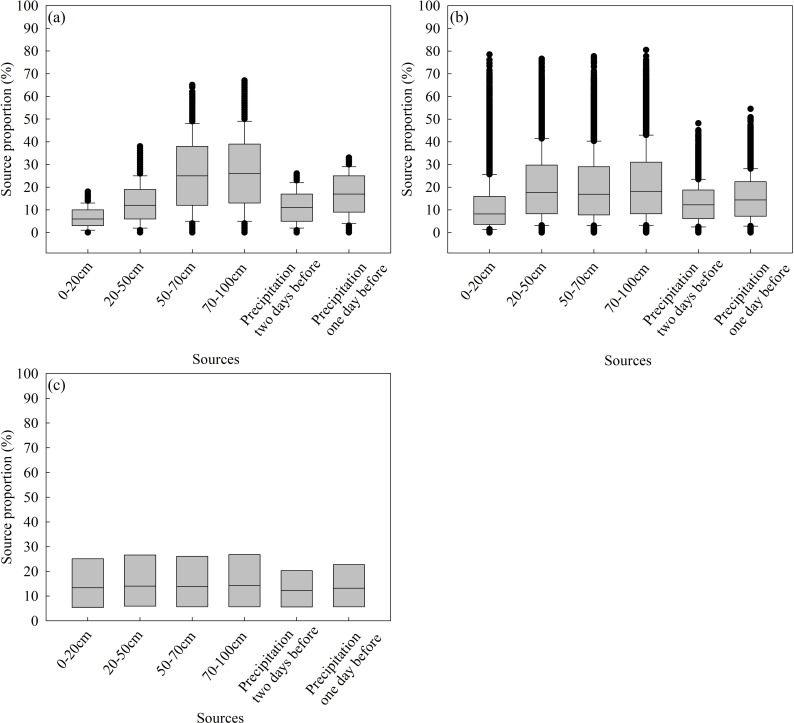
Box plot of water source proportional contributions of *C. korshinskii* using different models (a - IsoSource, b - MixSIR, and c - MixSIAR).

Fig 2 showed the dual isotope plot of the water samples, “mean value” referred to the arithmetic mean of replicates of the same water sample. The calculation results of the three models were shown in Table 3 and Fig 3. The results indicated that the proportional contribution of each water source performed by three models were different: the calculation results obtained by IsoSource and MixSIR were relatively similar, while the results obtained by MixSIAR were significantly different.

**Table 3:** Comparison of results for water source proportional contribution of *C. korshinskii* using different models.

Model		Result	Proportional contribution (%)					
0–20 cm	20–50 cm	50–70 cm	70–100 cm	Precipitation two days before sampling	Precipitation one day before sampling
**Linear model**	IsoSource	Upper quartile	3	6	12	13	5	9
		Median	6	12	25	26	11	17
		Lower quartile	10	19	38	39	17	25
**Bayesian model**	MixSIR	Upper quartile	3.64	8.28	7.84	8.31	6.11	7.16
		Median	8.27	17.59	16.93	18.19	12.24	14.43
		Lower quartile	15.95	29.77	29.04	31.01	18.75	22.38
	MixSIAR	Upper quartile	5.4	5.9	5.7	5.7	5.6	5.7
		Median	13.4	14	13.9	14.2	12.2	13.2
		Lower quartile	25.1	26.6	26.1	26.8	20.3	22.7

The results of IsoSource showed that, among the water sources used by *C. korshinskii*, the 70–100 cm soil water (median = 26%, short for the median of its proportional contribution was 26%) and 50–70 cm soil water (median = 25%) accounted for the largest proportional contribution (both the median, the upper and lower quartiles were the largest), and then followed by the precipitation one day before sampling (median = 17%), 20–50 cm soil layer (median = 12%), precipitation two days before sampling (median = 11%), and 0–20 cm soil water (median = 6%). Thus, the *C. korshinskii* mainly utilized the middle and deep soil water, and made little use of the surface soil water even if there occurred precipitation events two days before sampling.

The calculation results of MixSIR showed that the largest proportional contribution was 70–100 cm soil water (median = 18.19%), followed by 20–50 cm (median = 17.59%) and 50–70 cm (median = 16.93%), the rest were precipitation one day before sampling (median = 14.43%), two days before sampling (median = 12.24%), and 0–20 cm soil water (median = 8.27%), respectively. This result verified the conclusion derived from IsoSource that the *C. korshinskii* mainly utilized the middle and deep soil water, and rarely utilized surface soil water. In general, among the proportional contributions of different water sources, the largest contribution was 70–100 cm, and the smallest contribution was 0–20 cm, this conclusion drawn by IsoSource and MixSIR were consistent.

However, the results obtained by MixSIAR were significantly different from the previous two models, and the proportion contribution of all water sources were close, which indicated that the *C. korshinskii* used almost the same proportion of each layer soil water and precipitation. In terms of the median of proportional contributions, the difference between the largest contribution for the 70–100 cm soil water (14.2%) and the smallest contribution for precipitation two days before sampling (12.2%) was only 2%.

**Fig 4:**
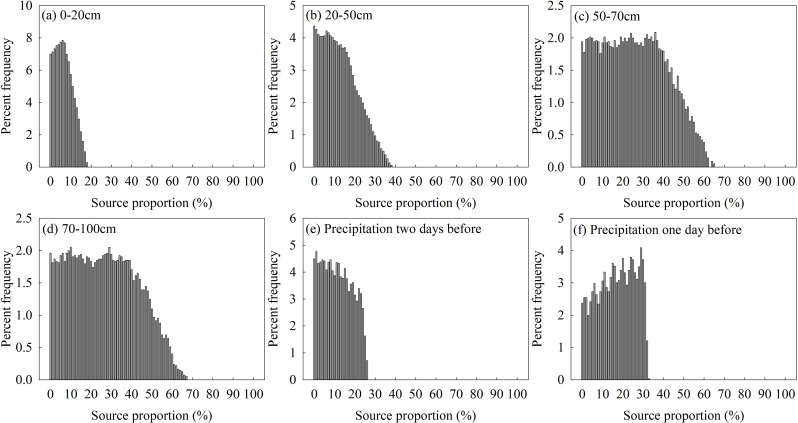
Percent frequency of contribution of different water sources to *C. korshinskii* using IsoSource.

**Fig 5:**
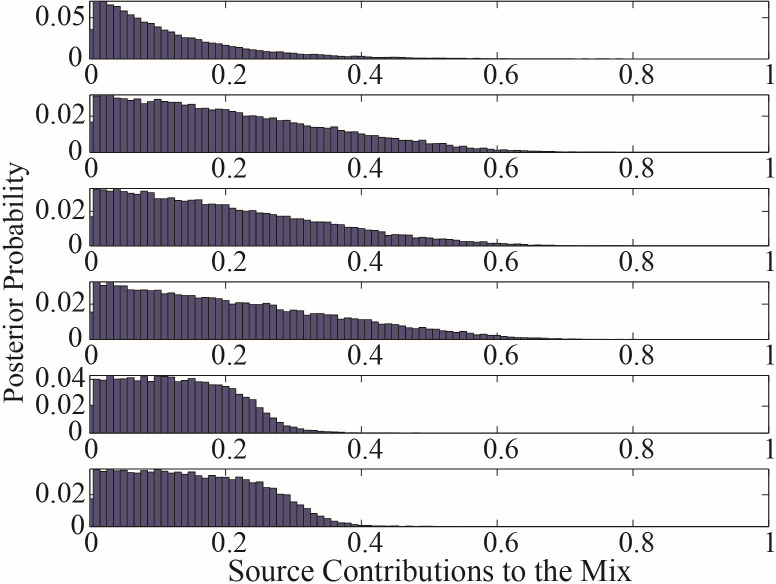
Posterior probability of contribution of different water sources to *C. korshinskii* using MixSIR.

**Fig 6:**
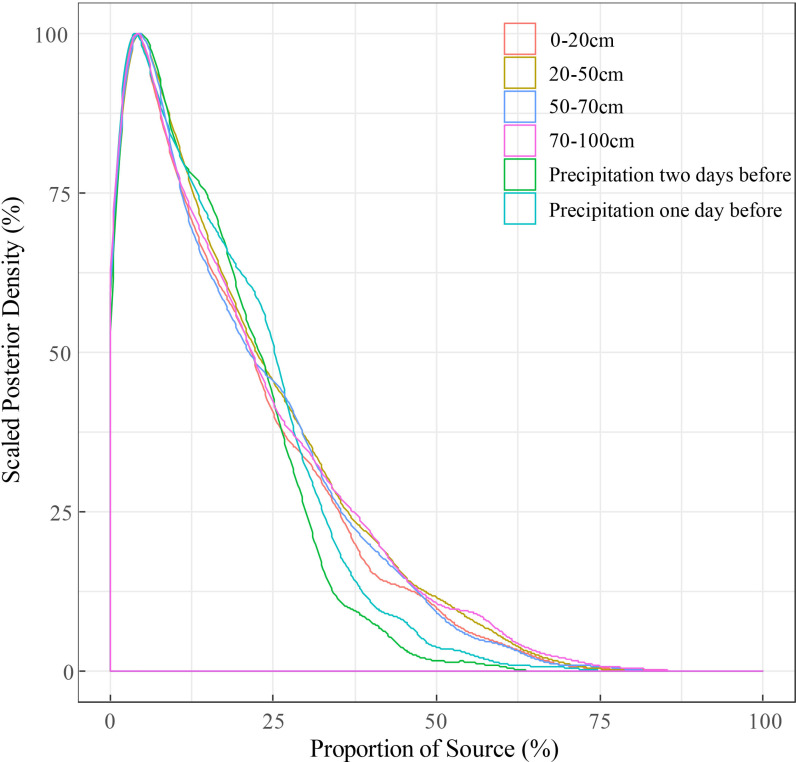
Scaled posterior density of contribution of different water sources to *C. korshinskii* using MixSIAR.

Although there were differences in the proportional contribution of each water source calculated by three mixing models, the maximum contribution obtained by the three models was consistently the 70–100 cm soil water, so it can be determined that the *C. korshinskii* at the research area mainly used deep soil water. However, the proportional contribution of other water sources cannot be determined because of the differences in the principles and algorithms on which the models are based. In addition, Figs 4–6 showed the original results outputted by the IsoSource, MixSIR and MixSIAR, respectively.

## Discussion

About the comparison of the results in this research with those of other similar studies. Some studies showed similar results to this research: four models (IsoSource, SIAR, MixSIR and MixSIAR) were compared using apple trees of different ages on the Loess Plateau [21], the results showed that the inter-model differences varied with soil depths (e.g., 1–3, 3–6, and > 8 m). For example, in terms of the contributions of soil water of 0–1 m to xylem water, the four models can be classified into two groups, that is, IsoSource and SIAR, MixSIR and MixSIAR; the models in the same group had similar performances, but their performances were significantly different from the models of the other group. It highlights the complexity of uncertainties in plant water source partitioning, and suggests that the uncertainties should be further investigated for different climate and vegetation types. Another research [23] found that there were significant differences in calculating the water source proportion of the main dominant plants in the secondary forest of southwest karst regions between IsoSource and MixSIAR models. There are other findings that differ from those in this paper, such as no significant difference is found in plant water source apportionment by the three Bayesian mixing models except for individual months during the growing season in the semiarid Loess Plateau [22]. Above all, the research for the proportion of plant source water contribution is a very complex topic, because extraction approach, analytical method, extraction conditions, soil type, soil water content, artifact of analytical/post analytical procedure may affect the isotopic compositions. Even with identical soil types, extraction procedures and analytical methods, the isotopic compositions of soil water extracted by cryogenic extractions showed great variations among different laboratories [31]. Therefore, these factors mentioned above may cause the different results in this study in comparison to those from the previous studies.

Regarding the statistical test for the differences of the three models, according to the characteristics of data distribution, the calculation differences of the three models were analyzed by ANOVA, and the results showed that there was no statistically significant difference (*P* = 0.857). According to the calculation principles of the three models and their respective advantages and disadvantages, the calculation results should have differences, but they are not supported by statistical tests, we think this is due to the sample size is too small, moreover no statistical difference among the sample group is not equal to no statistical difference among the total, in the future we need to increase the sample size for further validation.

The differences in the results after incorporating prior information were not compared, only the results of different models on the same sample data were conducted. Considering prior information would get more accurate proportional contributions for water sources, the results may have some changes. In addition, the example data used in this research were shrubs, whether there will be different results between trees and herbs requires further research in the future.

## Conclusion

This research summarized and compared the basic information, advantages and disadvantages of three stable isotope mixing models using a typical plant *C. korshinskii* in northeastern Tibetan Plateau as an example. The differences of the calculation results based on measured hydrogen and oxygen stable isotope composition in plant xylem water and potential source water were compared. It was found that there were differences in the calculation results of three models: the results of IsoSource and MixSIR were similar, but the results of MixSIAR were quite different from the former two. Nevertheless, the largest contribution calculated by the three models was consistent. Thus, there were no model differences when determining the primary water source for *C. korshinskii* in northeastern Tibetan Plateau.
